# Direct-to-Consumer Genetic Testing on Social Media: Topic Modeling and Sentiment Analysis of YouTube Users' Comments

**DOI:** 10.2196/38749

**Published:** 2022-09-15

**Authors:** Philipp A Toussaint, Maximilian Renner, Sebastian Lins, Scott Thiebes, Ali Sunyaev

**Affiliations:** 1 Department of Economics and Management Karlsruhe Institute of Technology Karlsruhe Germany; 2 HIDSS4Health – Helmholtz Information and Data Science School for Health Karlsruhe/Heidelberg Germany

**Keywords:** direct-to-consumer genetic testing, health information, social media, YouTube, sentiment analysis, topic modeling, content analysis, online health information, user discourse, infodemiology

## Abstract

**Background:**

With direct-to-consumer (DTC) genetic testing enabling self-responsible access to novel information on ancestry, traits, or health, consumers often turn to social media for assistance and discussion. YouTube, the largest social media platform for videos, offers an abundance of DTC genetic testing–related videos. Nevertheless, user discourse in the comments sections of these videos is largely unexplored.

**Objective:**

This study aims to address the lack of knowledge concerning user discourse in the comments sections of DTC genetic testing–related videos on YouTube by exploring topics discussed and users' attitudes toward these videos.

**Methods:**

We employed a 3-step research approach. First, we collected metadata and comments of the 248 most viewed DTC genetic testing–related videos on YouTube. Second, we conducted topic modeling using word frequency analysis, bigram analysis, and structural topic modeling to identify topics discussed in the comments sections of those videos. Finally, we employed Bing (binary), National Research Council Canada (NRC) emotion, and 9-level sentiment analysis to identify users' attitudes toward these DTC genetic testing–related videos, as expressed in their comments.

**Results:**

We collected 84,082 comments from the 248 most viewed DTC genetic testing–related YouTube videos. With topic modeling, we identified 6 prevailing topics on (1) general genetic testing, (2) ancestry testing, (3) relationship testing, (4) health and trait testing, (5) ethical concerns, and (6) YouTube video reaction. Further, our sentiment analysis indicates strong positive emotions (anticipation, joy, surprise, and trust) and a neutral-to-positive attitude toward DTC genetic testing–related videos.

**Conclusions:**

With this study, we demonstrate how to identify users' attitudes on DTC genetic testing by examining topics and opinions based on YouTube video comments. Shedding light on user discourse on social media, our findings suggest that users are highly interested in DTC genetic testing and related social media content. Nonetheless, with this novel market constantly evolving, service providers, content providers, or regulatory authorities may still need to adapt their services to users' interests and desires.

## Introduction

### Background and Objectives

Since the completion of the human genome project in 2003, dwindling genome sequencing costs and a rising interest in genomics among the general public have paved the way for direct-to-consumer (DTC) genetic testing [1]. Today, users can purchase DTC genetic tests via the internet for less than US $100 to gain genetic insights into their health, traits, heritage, and more without the involvement of health care professionals [2]. By providing users with such interesting and novel insights, DTC genetic testing markets are growing continuously. For example, North America's DTC genetic testing market alone accounted for 39% of an estimated global market value of US $1.5 billion in 2021. Moreover, with a projected annual growth rate of 15.3%, the DTC genetic testing market value is expected to triple in the next 8 years [3].

The uprise of DTC genetic testing and self-responsible genetics has also sparked countless ethical, social, technical, and legal issues [1]. For example, critics argue that DTC genetic testing lacks clinical validity and meaningful interpretation of test results, whereas service providers can make unregulated advertising and marketing claims, especially for health-related tests [1,2,4-7]. Indeed, consumers taking multiple DTC genetic tests found themselves receiving different results depending on the service provider [8]. Another concern often discussed by researchers and consumers is the potential sharing and reselling of genetic data (eg, to pharmaceutical companies) and the resulting implications on genetic privacy, including genetic data access to insurance companies, employers, law enforcement agencies, or malicious entities like hackers [9-14]. Although many consumers perceive these practices as unfair, low prices and potential genetic insights often outweigh the aforementioned concerns [15]. However, due to genetic similarity, these consequences may also apply to blood relatives who were not involved or did not consent to genetic testing [13,16]. This also ties in with media and research reporting that consumers in the United States use DTC genetic ancestry tests to prove their “genetic purity,” leading to instances of racism and genetic discrimination on social media [17,18].

With the increasing spread and availability of DTC genetic testing [2] and a general tendency in society to retrieve as well as discuss health information and health-related topics on the internet [19], it is by no means surprising that DTC genetic testing is a frequent and recent topic on many social media platforms [18,20,21]. In particular, YouTube, one of the largest social media platforms and the most comprehensive web-based video platform [22], serves as the first port of call for many internet users to discuss health information and DTC genetic testing in particular [23]. While YouTube can serve to share health information and experiences with a big audience for content creators (eg, consumers, service providers, health care professionals, or journalists), it also enables user discourse through textual comments below individual videos [24].

Understanding the topics, opinions, and attitudes discussed by the users can prove crucial for many stakeholders, as comments are the main form of user reaction and feedback on social media [23]. Service providers may gain, for instance, insights into consumer demands, whereas content creators may improve their videos by adjusting their content to meet user preferences. Moreover, with the ongoing debate on ethical and legal concerns toward DTC genetic testing [1,7], user opinions are of utmost importance to regulation authorities, politicians, and the industry in general. However, many stakeholders lack the means to extract the core themes discussed and attitudes expressed in the comments sections effectively and efficiently, given the sheer number of comments and manifold writing styles of users.

Extant research regarding DTC genetic testing on social media confirms this lack of understanding. Prior research focuses on microblogging services such as Twitter [25,26], Reddit [27], or 4chan [18] to investigate user discourse on DTC genetic testing and shows that we are still puzzled about users' interests and opinions toward DTC genetic testing. Inconsistent findings regarding which topics users discuss on different platforms (eg, ancestry testing on Twitter [25] and health testing on Reddit [27]) suggest that the DTC genetic testing discourse varies from platform to platform and must thus be investigated separately. Moreover, research has already shown the value of analyzing users' opinions and attitudes through user comments from select platforms for DTC genetic testing–related content. For instance, Mittos et al [18] have uncovered extensive use of hate speech on Twitter, whereas Basch et al [20] have identified the need for educational content about genetic testing on TikTok. Few studies have investigated information about DTC genetic testing on YouTube while primarily analyzing the multimedia information (ie, the content of the videos) [28-31] and overlooking the textual information provided by users' comments (see Multimedia Appendix 1 for a complete overview of research on DTC genetic testing on social media). Because most users do not actively produce YouTube videos but only consume them, we believe that analyzing the topics that users discuss in the YouTube comments sections provides a new perspective on the ongoing discussion regarding DTC genetic testing–related videos on social media platforms. Consequently, we ask the following research questions (RQs):

RQ1: What topics do YouTube users discuss in the comments sections of DTC genetic testing–related videos?

RQ2: What are users' attitudes toward DTC genetic testing–related videos, as expressed in their comments on YouTube?

To answer our RQs, we analyzed the 248 most viewed videos dealing with DTC genetics in a 3-step exploratory approach. First, we analyzed the selected videos regarding media type, genetic test purpose, and related health information. Second, we employed topic modeling to investigate user discourse in the comments sections of those videos. Third, we conducted a sentiment analysis unveiling users' attitudes toward the discussed topics and DTC genetic testing videos in general.

Through our study, we contribute to research and practice in several ways. As for research, we add to the literature on user attitudes toward DTC genetic testing by delineating topics and opinions discussed about these genetic tests. Further, we contribute to the research stream regarding health information on social media by showing that YouTube comments provide valuable insights on user discourse on social media and demonstrate that DTC genetic testing and health information topics may generally vary from platform to platform. As for practice, our research may help providers of DTC genetic testing services and regulatory authorities gain further insights into user attitudes and consequently adapt or improve genetic testing services and regulations. As most videos are user-generated, our analysis of user discourse can provide valuable insights on the topics discussed in the comments sections of these videos, providing content creators with valuable information for improving their future DTC genetic testing–themed videos.

### Health Information on Social Media Platforms

During the past decade, social media platforms have become increasingly attractive in the digital health sector as a means of communicating medical information [32]. In addition to accessing professional and nonprofessional medical information, users can also share their experiences and get in touch with each other [33]. Users already discuss various health topics like diabetes, medication and medication information, physical health, mental health, cancer, or more recently, COVID-19 on social media [19,34-38].

Consequently, information dissemination platforms (see Multimedia Appendix 1 for a detailed description of social media platform types), such as YouTube, have garnered interest from researchers to study various health care–related topics. For example, studies have investigated users' attitudes toward the effect of sleep-aiding music [24], users' preferences regarding treatment and symptoms of diabetes as well as the social culture pertaining to diabetes-related video clips [39], or public opinions and concerns about daily coverage of the COVID-19 crisis in Canada [23].

### DTC Genetic Testing

DTC genetic testing differs from traditional clinical genetic testing in that it is initiated by the consumers and does not require the direct interaction of consumers with health care professionals [2]. With the internet being the leading advertising and distribution channel, the DTC genetic testing service provider usually sends a DNA sample collection kit (eg, buccal swab or blood spot collection) to the consumers' homes for self-collection [5] or arranges for sample collection at a local laboratory [7]. Afterward, the service provider may perform various genetic analyses and then return the results directly to the consumers via the internet or mail [5]. Regarding DTC genetic testing, the consumers can choose the interpreter (ie, service provider) and the type and objective of the analysis of their genetic information (as opposed to a health care professional interpreting the genetic data). The most common types of testing services offered include ancestry tests (eg, AncestryDNA), nonmedical lifestyle tests (eg, FitnessGenes), relationship tests (eg, EasyDNA), and health tests (eg, 23andMe) [2]. Although DTC genetic testing provides consumers with novel and valuable information, it also has its downsides, such as consumers being responsible for managing and ensuring the security of their personal genetic information [1].

## Methods

### Research Approach

**Figure 1:**
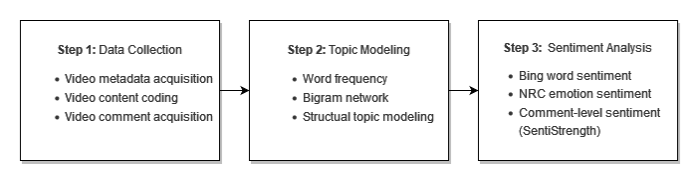
Overview of the 3-step research approach. NRC: National Research Council Canada.

We employed a 3-step exploratory research approach to answer our RQs (see Figure 1). First, we performed comprehensive data collection by gathering DTC genetic testing–related videos on YouTube, including their comments, and coding the contents of these videos. Second, we performed topic modeling for the user discourse in the comments sections to reveal topics discussed in those comments (answering RQ1). Third, we analyzed users' attitudes toward DTC genetic testing videos using sentiment analysis (answering RQ2).

### Data Collection

We used the official YouTube application programming interface (API) to create a list of the most relevant DTC genetic testing–related videos on YouTube. With the region set to the United States (ie, the largest DTC genetic testing market), we queried the 300 most viewed video results for each of 6 different DTC genetic testing–related search terms (ie, direct to consumer genetic testing, home genetic testing, ancestry testing, DNA testing, genetic testing, and 23andMe). Thereafter, we combined the 1800 results from the 6 queries, removed duplicates, and sorted them by video views in descending order. We further excluded all videos with less than 50,000 views because they had very few comments per video (average of 61.2), with many having no comments (n=336).

Next, the remaining 468 videos were reviewed for relevance through iterative manual inspection by 2 researchers, with a third researcher breaking ties in case of differences. For this, our predefined exclusion criteria were as follows: (1) videos not focusing on DTC genetic testing, (2) videos focusing on genetic testing of animals, (3) videos focusing on clinical prenatal genetic testing, (4) videos not in English, (5) live stream videos, (6) duplicate videos (ie, reuploads from different users), (7) videos commenting/reacting on videos (ie, showing the original video and adding commentary), or (8) videos with disabled ratings and comments sections (see Multimedia Appendix 2 for a detailed overview of the data collection process, including a rationale for each exclusion criterion). This resulted in a total of 250 relevant videos.

To gain insights on what topics the videos entailed, particularly the goal of the genetic test presented and the presentation type of the video, we coded the included videos according to their genetic test purpose and media type. For the genetic test purpose, we selected the most common test types suggested in the literature (ie, ancestry, traits, genetic predisposition, relationship, and other [2,7]). As for the media type, we adapted the categories used by Zhang et al [39] to our set of videos. Therefore, the categories were advertising, documentary, interview, news, user-generated video, and other. After the initial coding and comparison of 20 videos, 2 researchers conducted deductive coding of the remaining videos in parallel. In general, the agreement between both researchers was high, with the genetic test purpose and media type having Cohen κ values of 0.581 and 0.613, respectively. Differences in coding were discussed with a third author to break ties. This coding information allowed us to further analyze the comments regarding the contents of the videos and served as a base to evaluate the discussions in the comments.

With the final coded set of 250 videos in place, we again used the YouTube API to download each video's 500 most recent comments. This number was chosen due to the YouTube API download limitations while still allowing meaningful analysis. Among these, 80 videos had less than 500 comments, and 2 videos were no longer available, leaving us with 84,082 comments from 248 videos, which is a sufficient number for topic modeling and sentiment analysis [eg, 28,31,40,41].

### Topic Modeling of Comments

To answer our first RQ, we employed topic modeling to identify common topics discussed by users in the comments sections of DTC genetic testing–related YouTube videos. Topic modeling is frequently used in medical informatics and related disciplines for text mining large data sets (such as comments or tweets) and deducing meaningful topics [23,37,38,40,41]. For our study, we used several topic modeling approaches, including word frequency, bigram correlations, and structural topic modeling, as described and recommended by Silge and Robinson [42]. Because they are some of the most common topic modeling methods and include different approaches [42-44], they are well suited for our exploratory study design. All analyses and visualizations were conducted using R (version 4.1.0, R Foundation for Statistical Computing) in RStudio (version 1.4.1106) and the tidytext package (version 0.3.2).

Before conducting any topic modeling, we first separated the comments into 1-word tokens (ie, comments were split into single words) and performed 2 essential data cleaning tasks. First, we used the SnowballC package to perform word stemming. This step was necessary to ensure that words with identical meanings (eg, plural or verb) were grouped together to allow for meaningful topic modeling. For each word stem, the most frequent word was used to represent its stem (eg, test represents test, tests, test's, and testing). Second, we removed common stop words with the stop word list included in the tidytext package. This list comprises 1149 common stop words such as the, of, or to. As these do not hold any topical information, removing stop words reduces the data set size and benefits topic accuracy [42].

With the cleansed word list in place, we first conducted a word frequency analysis by grouping, counting, and listing the words in descending order. This provides an overview of the most used words and can give a first insight into topics discussed most prominently (eg, “DNA” occurs 15,702 times and “test” 10,902 times).

Second, we created word bigrams. We created a frequency list of 2-word tokens, which are found by pairing every 2 consecutive words in each comment (eg, “DTC genetic testing” results in the bigrams “DTC genetic” and “genetic testing”). In contrast to the single word list, bigrams can be used to span a network with the number of occurrences indicating the weight of each bigram edge [42]. To allow for meaningful interpretation, we found that setting a minimum of 70 occurrences resulted in a comprehensible network. Lower values led to the inclusion of less interpretable and impactful bigrams while cluttering the network (eg, “grocery store,” “hey kelsey,” or “omg lol”).

Finally, we conducted structural topic modeling with the help of the stm package [43]. Structural topic modeling aims to group words from different documents (ie, comments) into topics based on their co-occurrences [43]. The stm package uses document-level covariate information to estimate topic models for a given number of topics. We estimated models ranging from 15 to 100 topics in increments of 5. We then compared these models in terms of best-practice metrics, such as held-out likelihood, lower bound, residuals, and semantic coherence [42,45].

Although there is no definite answer for the correct number of topics [43], after a manual review of these metrics and discussion among 3 researchers, we selected 50 as the appropriate number of topics. A more detailed description of the structural topic modeling process and metrics, as well as a comparison with the 45- and 55-topic model, can be found in Multimedia Appendix 3.

With the 50-topic model chosen, we sorted topics according to prevalence and within each topic, the words contributing to it in descending order. We then manually inspected the 50 most prevalent topics and their 10 most contributing words to deduce meaningful topics and categorized them according to their content. For this, we relied on our prior knowledge of DTC genetic testing as well as knowledge on the content of the videos that we gained during the video coding phase of the data collection step. All topic assignments were discussed among 3 researchers.

### Sentiment Analysis of Comments

Because topic modeling can only help us identify topics discussed in the comments but not users' attitudes toward the videos, we next conducted word- and comment-level sentiment analyses to answer our second RQ. Sentiment analysis is a common tool to elicit people's opinions, sentiments, emotions, and attitudes from written language [46]. Although sentiment and attitude are near equivalents and often used synonymously, they do differ in the sense that sentiment is a more permanent disposition to react emotionally, cognitively, and conatively, whereas attitude is a disposition to react with belief, thought, feeling, and overt behavior as part of a larger sentiment [47]. In this sense, we can only deduce users' attitudes from a single YouTube comment and not their whole sentiment toward a certain topic.

Therefore, we decided to conduct 2 word-level sentiment analyses and 1 comment-level sentiment analysis to deduce users' attitudes. For the word-level sentiment, we again used the tidytext package, which entails typical word-level approaches that are well suited for a first exploratory overview [42]. We then followed an approach similar to that used by Mittos et al [18] for the comment-level analysis, who also performed sentiment analysis in the DTC genetic testing context.

Consequently, we first conducted a positive and negative sentiment analysis using the Bing lexicon, which consists of approximately 6800 words that are predefined and classified as either positive or negative [48]. Subsequently, we aggregated the sentiments by word and overall sentiment. Even though this method provides a good sentiment overview, the lexicon's limited number of words omits most topic-specific words.

We also used the National Research Council Canada (NRC) emotion lexicon to get a more detailed overview of users' sentiments toward DTC genetic testing [49]. This lexicon attributes 1 or multiple emotions to approximately 14,000 words (ie, a word may have more than 1 emotion), whereby the classification is also predefined. The emotions covered are anger, anticipation, disgust, fear, joy, sadness, surprise, and trust. Similar to the Bing lexicon, we classified and aggregated all words by NRC sentiment. However, initial inspection revealed that the terms “black” and “white” were strongly associated with negative and positive emotions, respectively. Because it was likely that the overproportional use of these words in our data set was due to ancestry testing–related topics, and to avoid a strong association of ethnicity with emotions, we reran the analysis without them.

For the comment-level sentiment analysis, we used SentiStrength [50], a Java-based sentiment tool optimized for short social web text in English such as Twitter tweets or YouTube comments. The tool reports 2 predefined and experience-based sentiments for each document (ie, comment). First, a negative sentiment ranging from –1 (not negative) to –5 (extremely negative) and a second, positive sentiment ranging from 1 (not positive) to 5 (extremely positive). When combining both, we obtained a total sentiment score between –4 and +4. After calculating the sentiment score for each comment, we performed several analyses regarding sentiment as well as media type and test purpose.

### Ethical Considerations

Ethics approval was not necessary for this study, as it did not directly involve human participants. All data used in this study (ie, videos and video comments) were publicly available on YouTube and accessible through the YouTube API at the time of retrieval. All results are only published in aggregated form, and single references are presented anonymously and without context to protect the privacy of the comments’ authors.

## Results

### Overview of Video Contents and Comments

We examined a total of 248 videos related to DTC genetic testing, collected on September 14, 2020, with a total of 30 videos from official company accounts (21 videos from 23andMe, 8 videos from Ancestry.com, and 1 video from MyHeritage). Based on the media type, these included 27 advertising-related videos, 14 documentaries, 16 interviews, 12 news, 174 user-generated videos, and 5 with other media types (mainly recordings of television shows such as The Late Show with Stephen Colbert or The Jim Jefferies Show/Comedy Central). Among the 248 videos, 194 videos address ancestry as a test purpose, 15 address trait testing, 9 address genetic predispositions, 19 address relationship testing, and 11 address other purposes (such as how to use a test kit or comparison/presentation of multiple genetic test purposes). In total, the videos had 724,574 comments on the day of video data aggregation. We collected the comments of the videos on January 3, 2021, focusing on the 500 most recent comments of each video (total number of comments=84,082). An overview of the video metadata, content, and comments is provided in Table 1.

**Table 1:** Overview of video metadata, content, and comments.

Video characteristic		Value
Number (N)		248
Date of collection		September 14, 2020
**Media type (n)**		
	Advertising	27
	Documentary	14
	Interview	16
	News	12
	User-generated videos	174
	Other	5
**Test purpose addressed (n)**		
	Ancestry	194
	Traits/characteristics	15
	Genetic predisposition	9
	Relationship	19
	Other	11
**Upload date**		
	Oldest	January 15, 2015
	Newest	July 7, 2020
**View count**		
	Minimum	52,802
	Maximum	20,453,890
	Average	1,158,064
**Likes**		
	Minimum	0
	Maximum	368,294
	Average	22,114
**Dislikes**		
	Minimum	0
	Maximum	10,277
	Average	813
**Duration (minutes)**		
	Minimum	00:31
	Maximum	34:23
	Average	09:30
**Comments**		
	Minimum	2
	Maximum	24,523
	Average	2922
**Comment publication date**		
	Oldest	March 29, 2017
	Newest	January 2, 2021

### Topics of the DTC Genetic Testing Video Comments

Word frequency analysis using the comments on DTC genetic testing–related videos provides valuable insights into the topics discussed by users. DNA (n=15,702), test (n=10,902), and people (n=9259) are by far the most frequent terms, thus indicating that users indeed primarily discuss DTC genetic testing in their comments. Additionally, we identified many words referring to ancestry testing such as ancestry (n=5015), african (n=6268), or american (n=6139). Moreover, words such as family (n=5252), dad (n=2932), or parents (n=2228) can be attributed to relationship tests. Overall, the 100 most frequent words resemble the test purposes identified from the videos themselves as well as a general excitement for DTC genetic testing videos (eg, video, n=4794; love, n=4751). Table 2 provides an overview of the 20 most frequent words. Additionally, Multimedia Appendix 4 provides a word cloud and overview of the 100 most frequent words.

The bigram network of the comments provides a more fine-grained picture of the words used together often. Unlike the single word cloud, it allows us to see how multiple words are connected. Additionally, the arrows indicate in which order the words appear, whereas the shade of the edge represents the frequency of the word pair. Therefore, we can deduce possible topics discussed by users from the network.

**Figure 2:**
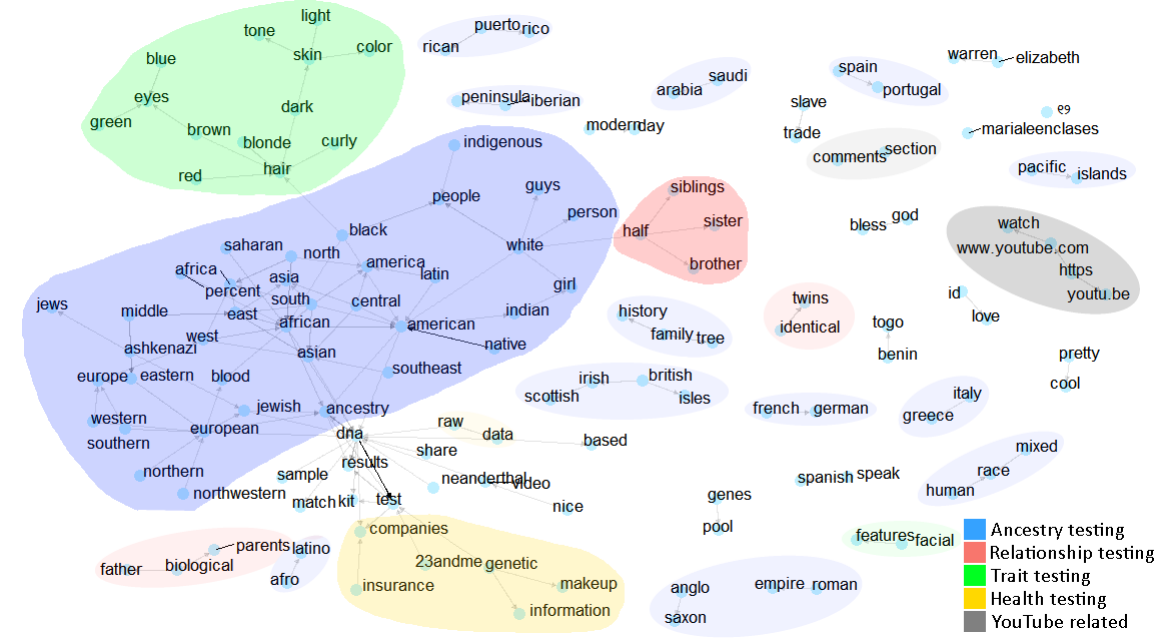
Bigram network of 2-word tokens found in the comments of direct-to-consumer genetic testing–related videos on YouTube with a minimum of 70 occurrences. Colored legends indicate topic attribution.

As shown in Figure 2, we identified 5 main topics within the network. The largest topic we identified revolves around ancestry testing (blue cluster). Although the most indicative bigram is “ancestry DNA” (n=679), most bigrams in this topic describe a specific heritage such as “native american” (n=3255), “north african” (n=831), or “middle eastern” (n=756), further substantiating that users largely discuss ancestry results of genetic testing in the comments. The second-largest topic deals with trait testing (green cluster) and holds bigrams such as “blonde/brown/red hair” (n=203/n=72/n=41), “skin color” (n=131), or “blue eyes” (n=285). The third topic entails bigrams related to health testing (yellow cluster). Typical bigrams include “insurance companies” (n=121), “genetic makeup” (n=76), and “23andme test” (n=72). The last topic related to genetic testing indicates relationship testing (red cluster). It includes bigrams such as “identical twins” (n=231), “half sister” (n=124), or “biological parents” (n=74). We also identified 1 topic not specific to DTC genetic testing but YouTube as a platform in general (gray cluster). The bigrams found in this topic are parts of video URLs, for example, “https youtu.be” (n=246) or “www.youtube.com watch” (n=201). This indicates that users often share videos in the comments sections of videos, possibly on related topics.

**Figure 3:**
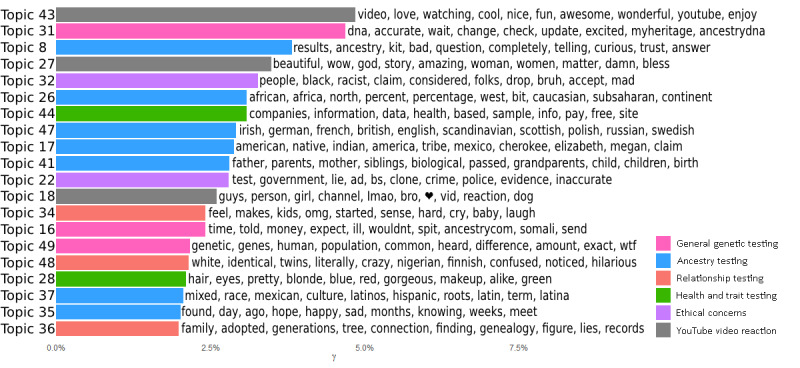
Top 20 topics and their 10 most indicative words from the 50-topic model. Colored legends indicate topic attribution.

Finally, we trained structural topic models, of which we selected the 50-topic model. Figure 3 shows the 20 most prevalent topics, including the 10 most important words for each topic of this model. The complete list of all 50 topics can be found in Multimedia Appendix 3. For a better overview of the topics discussed in the comments sections, we grouped these 20 topics into 6 categories, briefly described in the following:

**Table 2:** List of the 20 most frequent words obtained from comment analysis.

Rank	Word	Frequency (n)
1	dna	15,702
2	test	10,902
3	people	9259
4	african	6268
5	results	6178
6	american	6139
7	family	5252
8	european	5142
9	ancestry	5015
10	video	4794
11	love	4751
12	native	4665
13	white	4489
14	black	4203
15	lol	3469
16	asian	3276
17	irish	3177
18	mixed	2984
19	dad	2932
20	father	2782

#### General Genetic Testing

This topic group indicates a general interest in DTC genetic testing (eg, topics 16, 31, 49), entailing company names such as MyHeritage, AncestryDNA, or Ancestry.com and words of interest (eg, excited or expect). Moreover, topic 16 touches on the home collection (spit, tube) and financial (money) aspects of DTC genetic testing.

#### Ancestry Testing

In line with our previous findings, most topics are about the results of genetic ancestry testing. Topic 8 shows a general interest in ancestry testing by users. Topics 17, 26, 37, and 47 describe findings on heritage from a specific region, whereas topic 41 is about paternal and maternal ancestry. Additionally, topic 19 might indicate that users hope to find lost relatives through ancestry testing.

#### Relationship Testing

We also identified 3 topics about genetic relationship testing. Topics 34 and 48 deal with relationships between children such as identical twins, whereas topic 36 entails the aspects of adoption and genealogy (ie, searching for one's biological family).

#### Health and Trait Testing

Although less prevalent, health genetic testing and trait testing are also covered in the top 20 topics. Topic 44 focuses on health information and data, whereas topic 28 entails words on traits such as hair or eye color.

#### Ethical Concerns

The 50-topic model also reveals some topics not contained in our previous findings. Topic 32 touches on instances of racism signified through words such as black, racist, or mad. Given the ongoing and complex debate toward instances of racism in the United States and the majority of DTC genetic testing revolving around ancestry and heritage, this could explain why this topic was found in the comments of these videos. Moreover, topic 22 deals with users' concerns regarding genetic testing and the government, with words such as lie, ad, or crime.

#### YouTube Video Reaction

In contrast to the previous findings, topics 18, 27, and 43 do not directly relate to genetic testing but rather entail reactions to the videos on YouTube (eg, love, awesome, watching, video, or channel). Further, users seem interested in personal stories (eg, amazing, story, or reaction).

### Comparison of Topic Modeling Approaches and Identified Topics

Although the bigram network and structural topic modeling use different approaches, the majority of the identified topics are present in both methods. Both approaches show strong indications of ancestry testing, relationship testing, trait testing, and health testing topics. Moreover, both methods led to the deduction of a YouTube or YouTube video–related topic. Table 3 compares the topics covered by the bigram network and structural topic modeling and lists some of the most indicative bigrams and words for each method, respectively.

**Table 3:** Comparison of identified topics using the bigram network and structural topic modeling.

Topic	Bigram network	Structural topic modeling
General genetic testing	N/A^a^	Myheritage; ancestrydna; ancestrycom; excited; expect; spit; tube; money; genes; dna; genetic
Ancestry testing	Ancestry dna; native american; north african: middle eastern	Ancestry; african; american; native; irish; german; french; father; parents; race; mexican
Relationship testing	Identical twins; half sister; biological parents	Kids; cry; family; adopted; genealogy; lies
Trait testing	Blonde/brown/red hair; skin color; blue eyes	Hair; eyes; blonde; blue; red
Health testing	Insurance companies; genetic makeup; 23andme test	Companies; information; health; pay
Ethical concerns	N/A	Black; racist; claim; government; clone; crime; evidence
YouTube-related	https youtu.be; www.youtube.com watch	N/A
YouTube video reaction	N/A	Love; awesome; watching; video; channel; amazing; story; reaction

^a^N/A: not applicable.

### Sentiments of DTC Genetic Testing Video Comments

**Figure 4:**
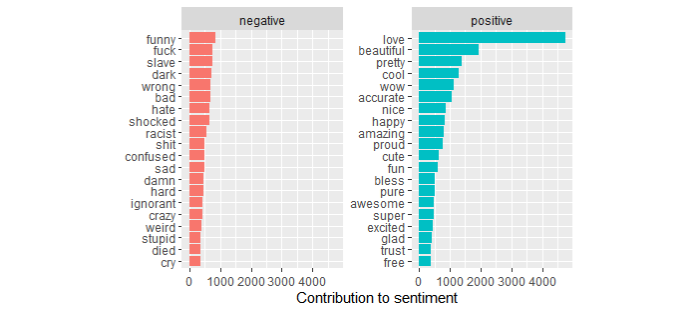
Bing sentiment by most frequent words for negative and positive sentiments.

Even though topic modeling can help unveil what users discuss in the comments sections, it does not provide insights into users' attitudes toward these topics. Therefore, conducting a Bing sentiment analysis can provide a first overview of the sentiment regarding words used in the comments sections. Figure 4 shows the 20 most used words with negative and positive sentiments. The results show that the most used positive words are used significantly more often. In fact, the first negative word, funny (n=864), is only the seventh most used word overall in the sentiment list. Moreover, the positive word love (n=4751) is used overproportionally, having more than twice as many occurrences as the second most used word, beautiful (n=1953). However, when observing all positively and negatively classified occurrences, we can identify more negative word uses (n=38,734) than positive ones (n=35,897).

**Figure 5:**
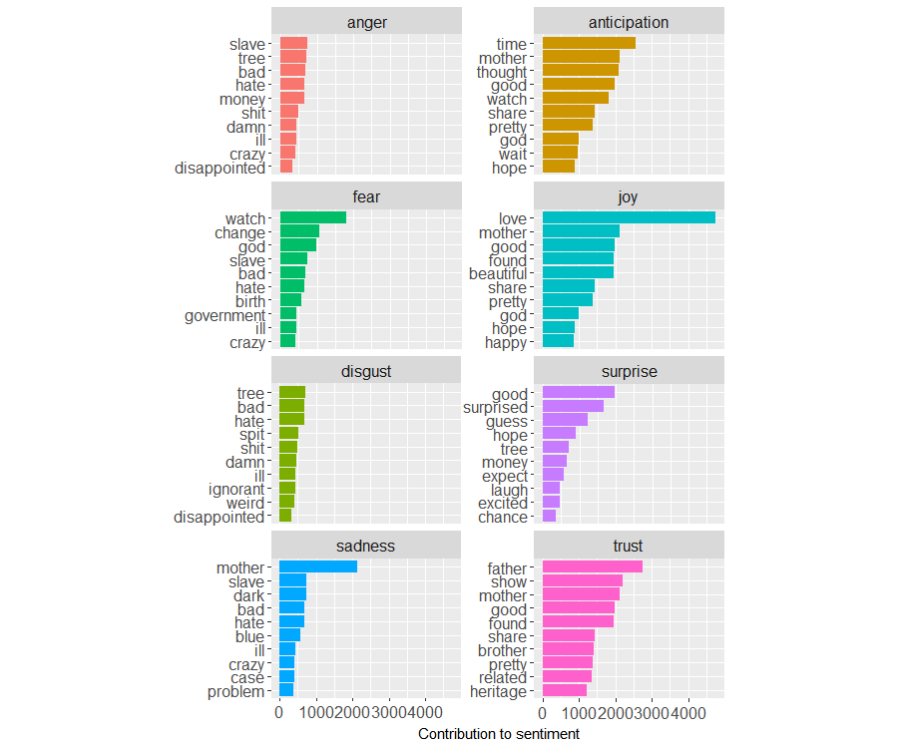
National Research Council Canada (NRC) sentiment by most frequent words for the emotions anger, anticipation, disgust, fear, joy, sadness, surprise, and trust.

Another type of sentiment analysis is the identification of emotions with the NRC lexicon. Our results show that the most frequent words representing positive emotions, namely anticipation, joy, surprise, and trust, have higher occurrences than the words expressing negative emotions, namely anger, fear, disgust, and sadness (see Figure 5). This finding is also supported by overall occurrences of positive word emotions (n=148,791) and negative word emotions (n=76,761). Love*,* the single most used word (n=4751), is associated with the emotion of joy, and the most frequent emotion is trust (n=54,814). In contrast, disgust (n=15,541) has the least word occurrences.

The comment-level sentiment analysis provides insights into user attitudes as well as attitudes toward DTC genetic testing videos and their respective content (ie, test purpose and media type). Although the SentiStrength sentiment can vary on a scale of –4 to 4, the average sentiment score of all comments is 0.32, meaning slightly positive. This is also reflected by almost half of all the comments (n=36,804) having a neutral sentiment (ie, 0). Grouping comment sentiment by video shows that the lowest sentiment score per video comments section is –0.62, whereas the highest is 1.33. Overall, only 30 of the 248 inspected videos have a negative sentiment, indicating an overall positive attitude toward DTC genetic testing videos.

**Figure 6:**
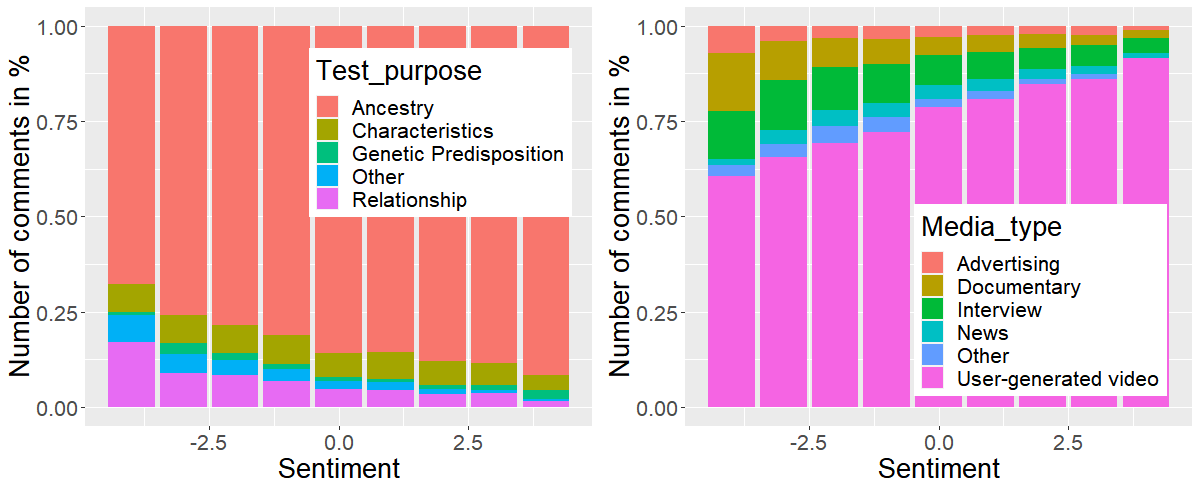
Spreads for test purpose (left) and media type (right) by sentiment.

When comparing comment sentiment regarding the test purpose of the videos, our results show that from the comments with a sentiment score of 4, 91.6% (230/251) are in the comments sections of videos about ancestry testing (most frequent test purpose), whereas for comments with a sentiment score of –4, ancestry testing videos only account for 67.9% (76/112). In contrast, only 1.6% (4/251) of the comments with a sentiment score of 4 are in the responses to a video dealing with relationship testing. However, this increases to 17% (19/112) for comments with a sentiment score of –4. As shown in Figure 6 (left), videos with an ancestry test purpose seem to evoke more positive user comments, whereas this is the opposite for relationship test videos.

The analysis of comment sentiment regarding media type unveils that user-generated videos account for the most significant number of positive comments with 91.6% (230/251) for a sentiment score of 4. On the contrary, for a sentiment score of –4, user-generated videos only account for 60.7% (68/112) of the comments. Consequently, as shown in Figure 6 (right), user-generated videos tend to evoke the most positive attitude toward their video content. This is in contrast to the media types advertising, documentary, and interview; all of these show an increase in the number of comments with decreasing sentiment values. For example, the number of comments for the media type documentary increases from 2% (5/251) with a sentiment score of 4 to 15.2% (17/112) with a sentiment score of –4. Therefore, advertisements, documentaries, and interviews may evoke more negative responses than user-generated videos.

## Discussion

### Principal Findings

Our analysis of user comments on DTC genetic testing–related YouTube videos yields several valuable findings. The test purposes found in the videos largely resemble the most common genetic test purposes, with most videos talking about ancestry or relationship testing and fewer about trait and health testing. This finding is in line with previous research on YouTube videos related to DTC genetic testing [28,31] and social media in general [20,21,25]. Nonetheless, in contrast to our study, Yin et al [27] found in their collected Reddit data set that relationship and health testing were more often mentioned than ancestry testing. Although Mittos et al [18] do not report the same finding for their Reddit data set, this may indicate that users of different social media platforms have other interests regarding DTC genetic testing. Another possible explanation for this could be that platform suggestion algorithms differ and may hence propose distinct content to users depending on the platform. Thus, discourses on the respective platforms should be investigated individually before assuming DTC genetic testing–related findings to be true across multiple platforms.

Moreover, most topics found with the bigram network and structural topic modeling can be attributed to common DTC genetic testing purposes. This indicates that user discourse revolves around the contents of the videos and DTC genetic testing. In line with previous research, we also identified topics dealing with general genetic testing and users' interest in and excitement for DTC genetic testing [18,51].

Besides, research has also shown instances of racism regarding ancestry testing on Twitter [18], which we also identified as a topic in the video comments. Even though it is unclear whether these comments relate directly to the content of the respective video or are in the replies to other comments, the identified topics largely revolve around racism and discrimination against African Americans and Native Americans. However, our results did not show any specific topics on the educational content of DTC genetic testing. Considering that consumers in the United States continue to use DTC ancestry tests to prove their “genetic purity” and discriminate against marginalized ethnic groups such as the aforementioned ones, especially on social media [17,18], research has called for more educational content and scientific explanations about DTC genetic testing [20,21]. Despite finding some videos expressing concerns toward DTC genetic testing (eg, documentaries), the majority of the videos seem to fail to highlight the advantages as well as the disadvantages and risks of DTC genetic testing. Hence, the discussions in the comments section may also largely neglect these aspects.

Sentiment analysis revealed that users have more negative attitudes toward the content of advertisements, news, or documentary videos compared to user-generated videos on DTC genetic testing. Although this finding could be explained through some media types being more thought-provoking (eg, documentaries covering disadvantages and risks of DTC genetic testing or news covering stories of genetic discrimination), another explanation might be that user-generated videos are often produced by single creators often trying to engage more with their YouTube community (eg, through specific content or active discussion in the comments sections) than, for example, a news broadcaster or DTC genetic testing service provider. Hence, this may result in a more positive user attitude. This assumption is further supported by our findings on YouTube-related and YouTube video reaction topics. On the one hand, these findings once again indicate that users discuss and respond to the content discussed in the respective videos, and on the other hand, they suggest a more complex discussion between content creators and their community (eg, through expressing enjoyment of content or including links to further YouTube videos). It should be noted that the revealed user attitudes on DTC genetic testing videos do not necessarily reflect user attitudes toward DTC genetic testing in general. However, as our topic modeling results suggest that user comments largely revolve around DTC genetic testing, it is likely that users’ attitudes toward DTC genetic testing videos also reflect their attitudes toward DTC genetic testing to some degree. This notion is further supported by the finding that videos discussing the disadvantages and risks of DTC genetic testing tend to have more negative user attitudes. Comparable results on user attitudes toward DTC genetic testing were also found for Twitter and related textual platforms [18,25,51], thereby strengthening this assumption.

Similar to DTC genetic testing–related Reddit posts [41], we found that user emotions toward DTC genetic testing videos expressed through the comments are mainly positive. The NRC sentiment and comment-level sentiment analyses also indicate a clear tendency toward a positive user attitude. This may be explained by the majority of videos being user-generated ones and aforementioned higher community engagement of content creators. Previous research on user sentiment toward Twitter tweets also shows a positive sentiment toward DTC genetic testing [51]. However, Mittos et al [18] found that most tweets only have a sentiment score of 0 or 1. In line with previous research [21,51], these less positive emotions and attitudes could indicate that although users are generally interested in DTC genetic testing, they still have reservations regarding this new technology. These reservations are mirrored in the results of the NRC sentiment analysis that highlighted fear as the most prominent negative attitude toward DTC genetic testing, whereas trust was the most prominent positive attitude. These reservations toward DTC genetic testing were also highlighted in prior research [7].

### Implications for Research and Practice

This study conveys several implications for research and practice. As for research, we contribute to the literature on user attitudes toward DTC genetic testing by investigating topics and opinions discussed about these genetic tests. We examined the 248 most viewed DTC genetic testing videos on YouTube in terms of their content (ie, test purpose, media type) and analyzed users' attitudes in the form of their comments. Further, we contribute to research regarding health information on social media by showing that YouTube comments provide valuable insights into user discourse on social media. This study suggests that video content and user comments are co-dependent and should therefore be investigated together. To this end, we provide new insights into the discourse on genetic testing on YouTube by showing that the discourse in the comments primarily revolves around the content of the videos. Our research indicates that the discourse on YouTube may differ from that on other social media platforms, and hence, a detailed and differentiated consideration of the different platforms may be necessary. We further contribute to knowledge regarding user behavior on social media by examining users' attitudes and emotions toward DTC genetic testing videos on YouTube.

As for practice, our research offers important implications for DTC genetic testing service providers, content creators, and regulatory authorities regarding user attitudes, which may help adapt or improve genetic testing services, multimedia content, or regulations. Similar to the study of Lee et al [21] involving Twitter, our identified topics indicate a lack of educational information about DTC genetic testing in YouTube videos. Further, sentiment analysis shows that users have more negative attitudes toward advertisements, news, or documentary videos and prefer user-generated content on DTC genetic testing. Hence, authorities could consider working with content creators to promote user education on DTC genetic testing. Finally, our topic modeling indicates instances of racism, especially regarding ancestry testing. Service providers and authorities should be aware of this and ensure genetic testing is not used for discrimination. Therefore, we suggest that it may be helpful to flag videos with high numbers of negative comments, including racism or anxiety, and provide further information regarding DTC genetic testing via banners or other visual cues, similar to those used on many platforms for content related to COVID-19 [52].

### Limitations and Future Research

The limitations of this study are as follows. First, we only considered a limited number of videos and comments. Even though we attempted to include an appropriate sample by saturating the videos and comments using metrics such as views and number of comments, examining all the initially identified videos (n=1325) and comments could provide further insight, particularly concerning topic modeling and sentiment analysis. Second, we limited our YouTube API queries to the United States because the related DTC genetic testing market is the most evolved there. However, other regions with striving markets, such as Asia [30], could offer further insights into user discourse and should therefore be investigated in future research. Third, because there is no way to determine the optimal number of topics [42], we concentrated on models in increments of 5, selecting the 50-topic model. Although adjacent models tend to have many similar topics, it is possible that we did not identify a vital topic covered in a different solution. Future research could also attempt using different topic modeling methods and larger sample sizes to unveil a more fine-grained view of the topics discussed. Fourth, despite covering several sentiment lexicons, they may have been limited with respect to words associated with a sentiment (eg, Bing sentiment), and research should further investigate YouTube comment sentiment to gain deeper insight into user attitudes. It should also be pointed out that the generic association of words with sentiment values and emotions could omit or alter some findings in specific contexts such as DTC genetic testing. However, we tried to minimize this effect by using different approaches and content-specific modifications such as removing the words “white” and “black” from the NRC sentiment analysis, as these were used overproportionally. Finally, although this study investigated videos spanning from 2015 to 2020, we did not specifically focus on whether or how user discourse and attitudes might have changed over time. Because we only collected the 500 most recent comments, the majority of these can be dated to 2021. However, the DTC genetic testing market has and continues to evolve and change rapidly [1,2,7,14]. Future research should thus consider a temporal analysis of DTC genetic testing videos and comments to investigate if the market changes also affected user discourse and attitudes.

### Conclusions

This study examined 248 DTC genetic testing videos and 84,082 comments on YouTube to investigate user discourse. To this end, we employed topic modeling and identified 6 prevailing topics discussed among users, which largely revolve around the test purposes mentioned within those videos, such as ancestry or relationship testing. Further, we conducted sentiment analysis, showing that users have positive emotions, as indicated by the NRC sentiments of anticipation, joy, surprise, and trust*,* and a generally neutral-to-positive attitude toward DTC genetic testing expressed through words such as love, beautiful, pretty, and cool as well as a positive attitude toward DTC genetic testing–related videos on YouTube in general. Through this study, we show how users' attitudes toward DTC genetic testing can be determined by analyzing topics and opinions based on YouTube video comments. Our findings show that users are highly interested in DTC genetic testing and related social media content. Nonetheless, with this novel market still evolving, service providers, content providers, or regulatory authorities may need to adapt their services to users' interests and desires.

## Appendix

Multimedia Appendix 1 Direct-to-consumer genetic testing on social media.

Multimedia Appendix 2 Data collection process.

Multimedia Appendix 3 Structural topic modeling.

Multimedia Appendix 4 Word frequency analysis results.

## Abbreviations

API: application programming interface
DTC: direct-to-consumer
NRC: National Research Council Canada
RQ: research question
