# Supplementary material for: Direct-to-Consumer Genetic Testing on Social Media: Topic Modeling and Sentiment Analysis of YouTube Users' Comments
Source: JMIR Infodemiology. 2022 Sep 15;2(2):e38749. doi: 10.2196/38749 (PMC10014090; doi:10.2196/38749)
Supplement: Multimedia Appendix 1 [file infodemiology_v2i2e38749_app1.pdf]

# Multimedia Appendix 1. Direct-to-Consumer Genetic Testing on Social Media.

Before conducting our study, we surveyed related literature on direct-to-consumer (DTC) genetic in social media. Because information on DTC genetic testing can be presented in multiple forms on social media (eg, text, video, audio), we first provide a brief overview of dominant types of social media platforms and thereafter show the results of our literature search:

## Types of Social Media Platforms

The wide field of social media platforms, in general, can be divided into three main types of platforms, namely networking sites (eg, Facebook), information dissemination platforms (eg, YouTube), and microblogging services (eg, Twitter) [32]. Information can be exchanged in different ways on these platforms (ie, textual or multimedia information), whereby the different types of social media platforms differ in how information is mainly shared [39]. Usually, information is primarily shared via one medium, but mixed forms of information are also possible. For example, networking sites (eg, Facebook) and microblogging services (eg, Twitter) typically rely on textual information sharing, although images or short videos may also be linked. Information dissemination platforms like YouTube, in turn, use multimedia information, where users can also engage in textual information exchange through comments [32].

## Research on Direct-to-Consumer Genetic Testing on Social Media

The increasing simplification and affordability of self-administered genetic testing, particularly ancestry testing, has led to an upsurge in users sharing and discussing health information about DTC genetic testing on social media [18] and has drawn the attention of researchers. Several studies have looked at DTC genetic testing on social media, for example, focusing on textual information from microblogging sites (eg, Twitter [26] or Reddit [18]). While research has examined the content and coverage of Twitter and similar services to understand the impact of social media on DTC genetic testing [eg, 27, 51], so far, few studies on DTC genetic testing have investigated YouTube as a platform for health information. Moreover, the limited studies available focus mainly on multimedia information in the form of video content [eg, 28, 29, 31]. To the best of our knowledge, Venkatesaramani et al [40] were the only ones to investigate the comments provided by the YouTube community in DTC genetic testing while focusing on the technical understanding of topic modeling for YouTube comments. However, striving to compare the performance of different topic modeling techniques and a rather small sample size of n=800 comments, they did not provide any insights into the comments' contents and what users discuss in these comments about DTC genetic testing. Table MA1-1 provides a complete overview of studies identified during our literature review.

Table MA1-1. Overview of research topics on DTC genetic testing on social media.

| **Paper** | **Year** | **Focus/Objective** | **Platform** | **Information type** | **# of instances** | **# of comments** |
|---|---|---|---|---|---|---|
| Basch et al [28] | 2020 | Popularity and content that is covered in videos | YouTube | Multimedia | 100 videos | - |
| Basch et al [20] | 2021 | Popularity and content that is covered in videos | TikTok | Multimedia & Textual | 100 videos | 77,498 |
| Chow-White et al [51] | 2018 | Content and sentiment toward 23andMe tweets | Twitter | Textual | 2075 tweets | - |
| Harris et al [29] | 2014 | Analysis of narrative storytelling in videos | YouTube | Multimedia | 20 videos | - |
| Kragh-Furbo et al [30] | 2017 | Content of 23andMe home collection kit unboxing videos | YouTube | Multimedia | 69 videos | |
| Lee et al [21] | 2020 | Popularity and the content covered in 23andMe tweets | Twitter | Textual | 1,000 tweets | - |
| Marcon et al [31] | 2021 | Content of ancestry testing results videos | YouTube | Multimedia | 117 videos | - |
| Mittos et al [25] | 2018 | Popularity and content that is covered in tweets | Twitter | Textual | 302,000 tweets | 269,292 |
| Mittos et al [18] | 2020 | Popularity and content that is covered in tweets or posts | Twitter, Reddit & 4chan | Textual | 386,170 tweets/posts | 280,012 |
| Roberts et al [26] | 2019 | Content on the authorization of DTC BRCA1/2 genetic testing | Twitter | Textual | 11,055 tweets | - |
| Venkatesaramani et al [40] | 2019 | Technical understanding of topic modeling on comments and detection of hate and offensive speech | YouTube<br>Twitter | Textual | -<br>24,802 tweets | 800 (YT)<br>- (Twitter) |
| Yin et al [27] | 2020 | Content of online board discussions | Reddit | Textual | 15,946 posts | 141,806 |
| This study | 2022 | Topic modeling and sentiment analysis of video comments | YouTube | Multimedia & Textual | 248 videos | 84,082 |
