# Supplementary material for: Direct-to-Consumer Genetic Testing on Social Media: Topic Modeling and Sentiment Analysis of YouTube Users' Comments
Source: JMIR Infodemiology. 2022 Sep 15;2(2):e38749. doi: 10.2196/38749 (PMC10014090; doi:10.2196/38749)
Supplement: Multimedia Appendix 2 [file infodemiology_v2i2e38749_app2.pdf]

Multimedia Appendix 2. Data Collection Process.

Figure MA2-1 outlines the data collection process for including direct-to-consumer (DTC) genetic testing-related videos on YouTube. Hereafter, we provide a more detailed explanation of the data collection process:

Figure MA2-1. Overview of the data collection process.

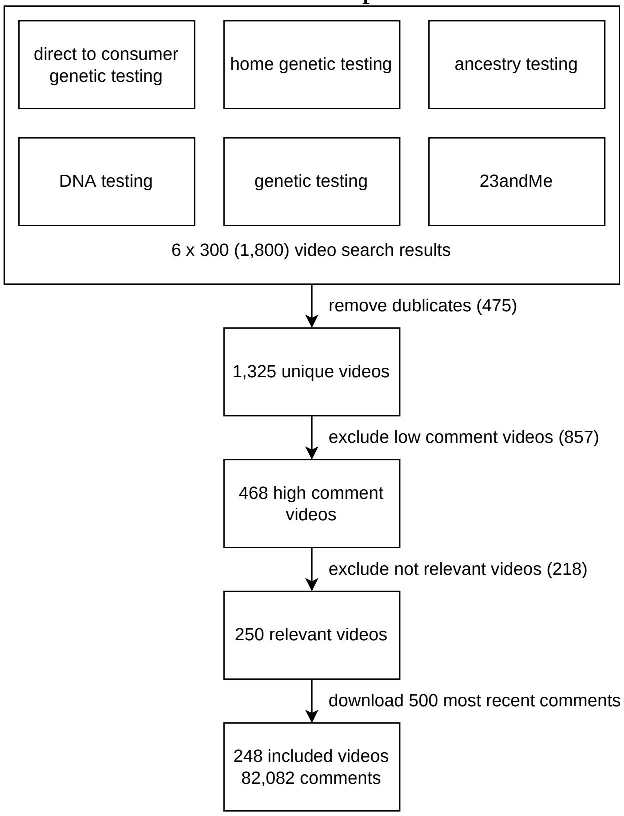

We utilized the official YouTube application programming interface (API) to create a list of relevant DTC genetic testing-related YouTube videos, which allows for automated search queries and data export. Because the DTC genetic testing market and usage of YouTube are most evolved in the United States, we set our search region to the United States. We then conducted six separate searches, retrieving the 300 most viewed video results for six different DTC genetic testing-related search terms. The terms used include variations of DTC genetic testing (ie, *direct to consumer genetic testing*, *home genetic testing*, and *ancestry testing*), general genetic testing (ie, *DNA testing* and *genetic testing*), as well as the largest company in the market (ie, *23andMe*). After that, we combined the 1800 results from the six queries, removed duplicates, and sorted them by video views in descending order, leaving 1,325 unique videos. Because topic modeling requires as much data as possible and because we wanted to have ca. 500 comments per relevant video, we decided to exclude videos with a low comment count before we conducted any further analysis. As our list showed that videos with less than 50,000 views had very few comments (an average of 61.2 comments per video, with 336 having none at all), we only considered videos with a view count greater than 50,000 views to ensure a sufficient number of comments to analyze. Thus, we excluded 857 videos.

Next, the remaining 468 videos were reviewed and selected for relevance, ensuring reliability by iterative manual inspection of two researchers, with a third researcher breaking ties in case of differences. For this, we predefined a set of exclusion criteria. These are (1) videos not focusing on DTC genetic testing, (2) video focusing on genetic testing of animals, (3) videos focusing on clinical prenatal genetic testing, (4) video is not in English, (5) live stream videos, (6) duplicate videos (ie, re-uploads from different users), (7) videos commenting on videos (ie, showing the original video and adding commentary), or (8) videos with disabled ratings and comments sections. The following Table MA2-1 provides an overview of the exclusion criteria used to select the 250 relevant videos for our study. Moreover, the table also provides a rationale for each criterion. In total, the analysis of relevance led to the exclusion of 218 videos and the inclusion of 250 videos with a potential of 724,574 comments, which is a sufficient size for topic modeling and sentiment analysis [eg, 28, 31, 40, 50].

Table MA2-1. Exclusion criteria and rationale for relevance analysis of videos.

**Exclusion Criterion**	**Rationale**
Videos not focusing on DTC genetic testing	Since this study aims at investigating YouTube videos about DTC genetic testing, we excluded all videos not or only marginally dealing with the topic.
Videos focusing on genetic testing of animals	Because this study strives to investigate user discourse and user attitudes toward DTC genetic testing of humans, we excluded all videos dealing with genetic tests targeted at animals.
Videos focusing on clinical prenatal genetic testing	Although prenatal genetic testing is often initiated by the consumer (ie, parents), clinical prenatal genetic testing does not strictly belong to DTC genetic testing as it requires the involvement of a healthcare professional. Moreover, it is only available to a limited target group (ie, future parents). Therefore, we excluded videos about clinical prenatal genetic testing.
Videos not in English	Since most YouTube search query results with the region set to the United States are in the English language, and other languages would prohibit meaningful investigation of the video's contents, we excluded all videos not in English. Moreover, videos in other languages are more likely to have comments in these languages as well, possibly impeding topic modeling and sentiment analysis, as the methods we applied are optimized for English words and sentences.
Live stream videos	Live stream videos mean recordings of YouTube live streams, usually with a runtime of one or more hours. Because these videos contain a conventional comments section and a live comments section (ie, comments will only be displayed at the same time as they were posted during the live stream), we excluded all live-stream videos. Moreover, these videos are usually unscripted and often focus on live interaction between the content creator and the viewers instead of presenting a prepared topic.
Duplicate videos	Duplicate videos are videos re-uploaded by other YouTube channels without alterations. Because these do not hold any additional content and thus information, we excluded all duplicate videos. Moreover, most of these re-uploads are illegitimate.

**Exclusion Criterion**	**Rationale**
Videos commenting on videos	Similar to duplicate videos, videos commenting on videos re-upload the videos created by others but provide their commentary (eg, in the form of a voice-over or pausing the original video). Although these videos may provide additional information, we found that they are an alternative form of reacting to and commenting on a video (ie, an audio/video instead of a written comment). Consequently, we decided to exclude videos commenting on videos as the comments in the comments section would also deal with information presented in the underlying video.
Videos with disabled rating and comments section	Because this study requires comments to analyze the user discourse in the comments section of DTC genetic testing-related videos, we excluded all videos with a disabled rating and comments sections, as these could not be further investigated.
