# Supplementary material for: Direct-to-Consumer Genetic Testing on Social Media: Topic Modeling and Sentiment Analysis of YouTube Users' Comments
Source: JMIR Infodemiology. 2022 Sep 15;2(2):e38749. doi: 10.2196/38749 (PMC10014090; doi:10.2196/38749)
Supplement: Multimedia Appendix 3 [file infodemiology_v2i2e38749_app3.pdf]

Multimedia Appendix 3. Structural Topic Modeling.

We conducted a structural topic modeling with the help of the stm package [43]. Structural topic modeling aims to group words from different documents (ie, in our case, comments) into topics based on their co-occurrences [43]. The stm package utilizes document-level covariate information to estimate topic models for a given number of topics.

Estimating the optimal topics for a given data set can be difficult. Therefore, the stm package provides for key decision metrics, namely held-out likelihood, lower bound, residuals, and semantic coherence. The held-out likelihood gives the probability of a word appearing within a document when that word has been removed from the document in the estimation step. Consequently, a good solution should have a rather high held-out likelihood. The lower bound indicates the number of iterations needed for the model to achieve convergence. Models that achieve convergence in fewer iterations (ie, a rather low lower bound) indicate a more stable and hence preferable solution. The residuals indicate the degree of overdispersion of the variance during the data generating process. Therefore, lower residuals indicate a more stable model. Finally, semantic coherence is the probability that words in a given topic frequently co-occur together. A high semantic coherence thus indicates a good solution. However, semantic coherence is also influenced by the number of topics in a model. Hence, solutions with fewer topics often tend to have higher semantic coherence [42, 45].

We first tested smaller numbers ranging from 5 to 15 topics. The metrics, however, quickly showed that there is a strong trend toward larger topic solutions. Therefore,
we estimated models ranging from 15 to 100 topics in increments of five. Figure MA3-1 shows the held-out likelihood, lower bound, residuals, and semantic coherence for these solutions.

Figure MA3-1. Structural topic modeling metrics for 15 to 100 topics.

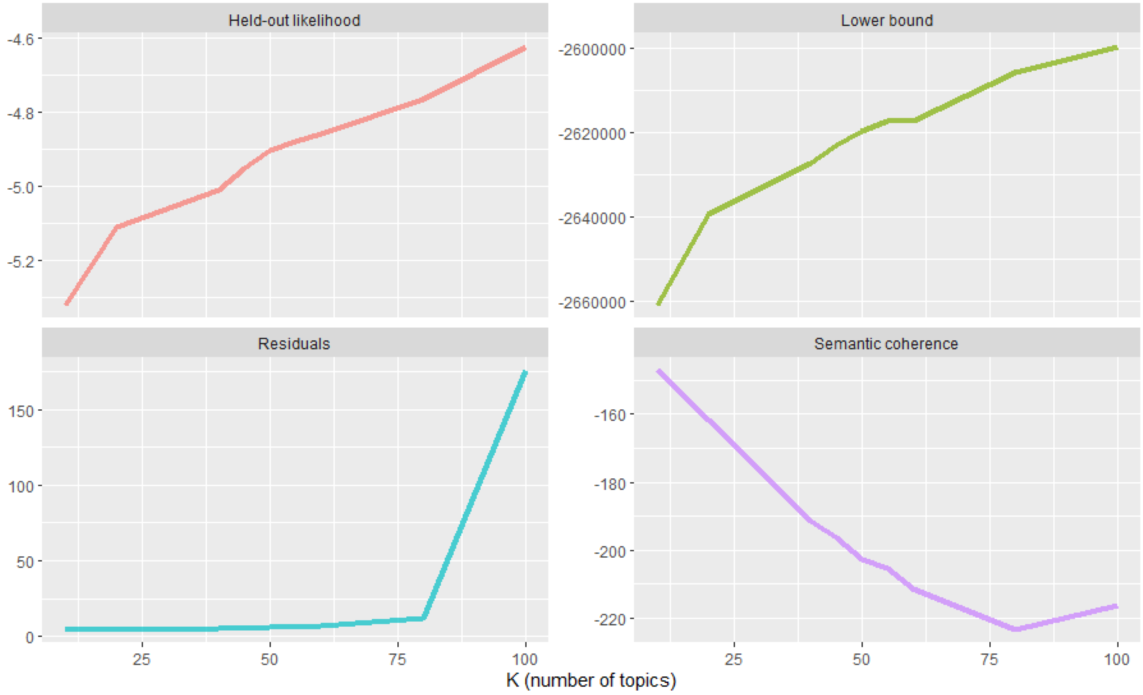

While there is no definite answer for the correct number of topics [43], as explained previously, high held-out likelihood, as well as semantic coherence (ie, how exclusive a word is to a topic) and low residuals and lower bound, may indicate a good possible number of topics. Manually reviewing these metrics within a discussion of three researchers, we determined 50 to be the appropriate size of topics.

With the 50-topic model chosen, we sorted topics according to prevalence (γ), and within each topic, the words contributing to it in descending order (see also Table MA3-1). We then manually inspected the 50 most prevalent topics and their ten most contributing words to deduce meaningful topics. Moreover, to ensure that the 50-topic model was the best solution, we manually inspected the 45-topic and 55-topic models. However, we found the 50-topic model to allow for a better deduction of topics. Figures MA3-2, MA3-3, and MA3-4 show the top 20 topics with their ten most contributing words in descending order of prevalence (γ) for the 45-, 50-, and 55-topic model solutions.

Figure MA3-2. Top 20 topics for the 45-topic model.

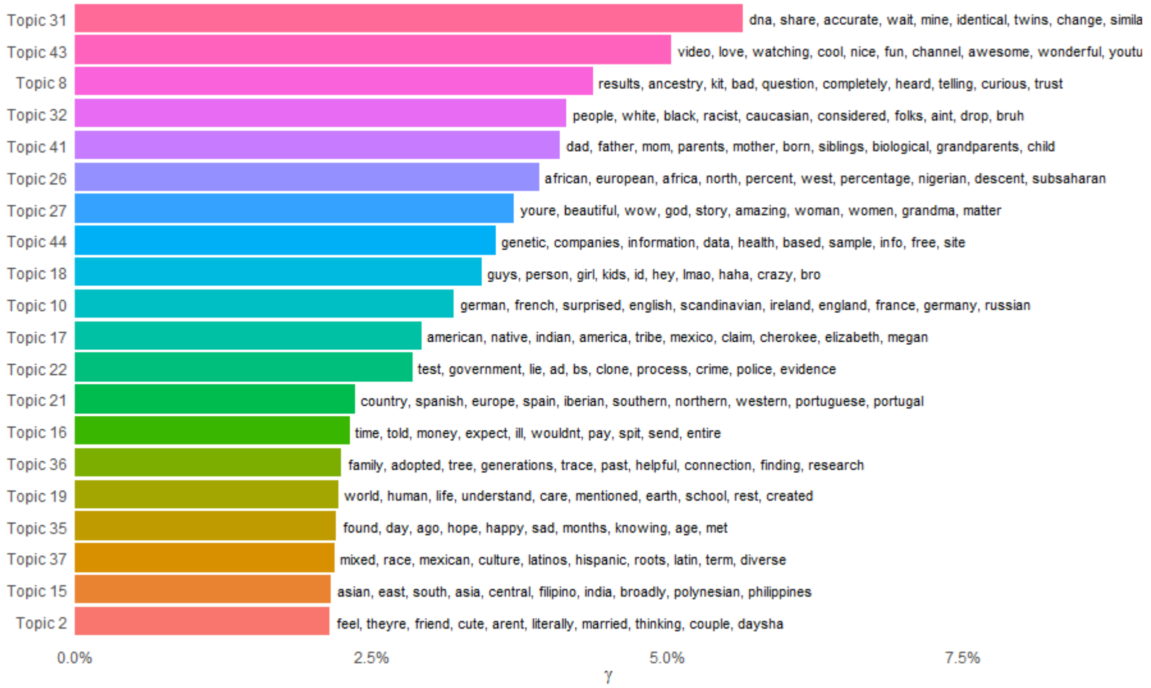

Figure MA3-3. Top 20 topics for the 50-topic model.

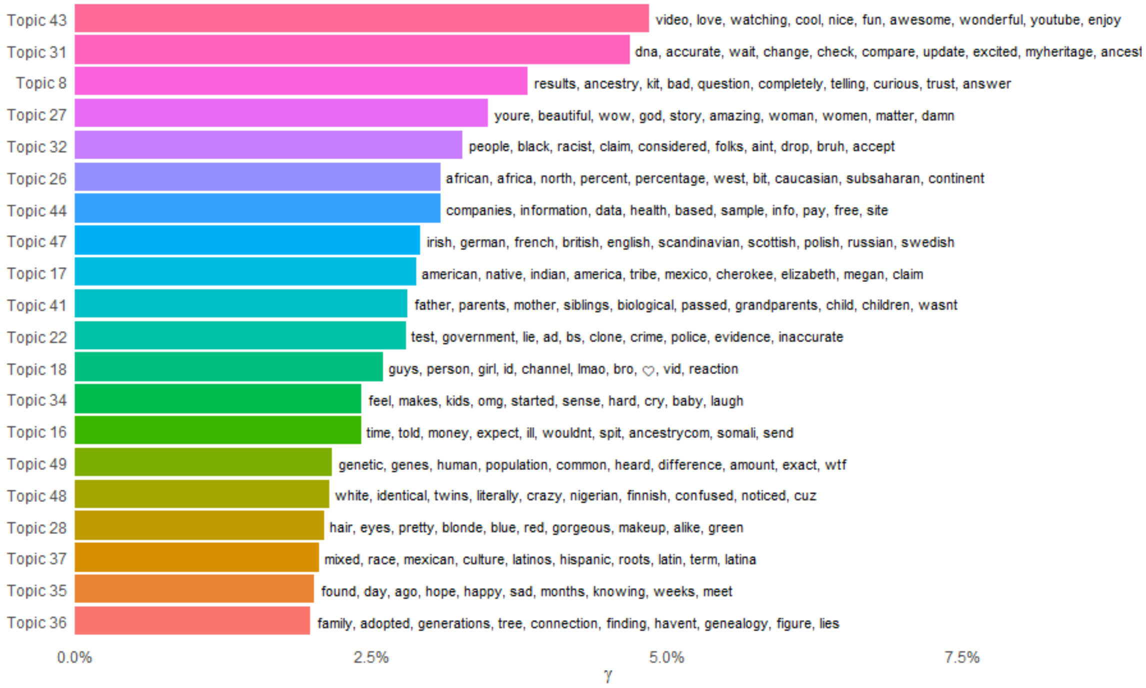

Figure MA3-4. Top 20 topics for the 55-topic model.

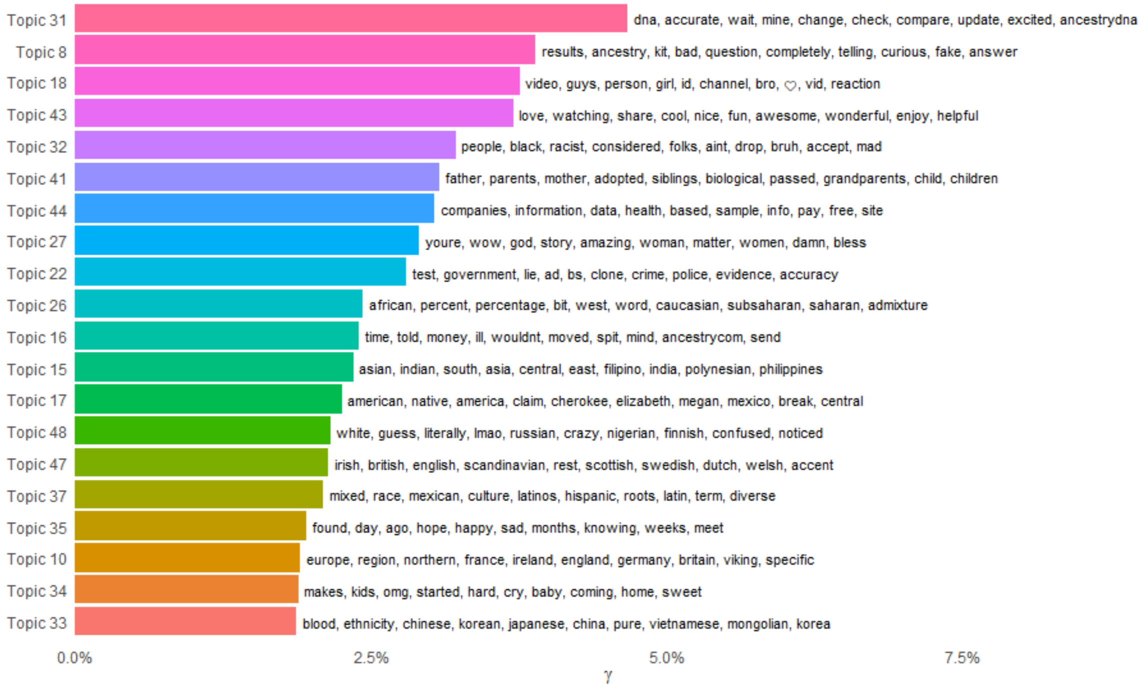

Table MA3-1. 50-topic model complete term overview.

ID	γ	Word 1	Word 2	Word 3	Word 4	Word 5	Word 6	Word 7	Word 8	Word 9	Word 10
43	.0485	video	love	watching	cool	nice	fun	awesome	wonderful	youtube	enjoy
31	.0468	dna	accurate	wait	change	check	compare	update	excited	myheritage	ancestrydna
8	0.038	results	ancestry	kit	bad	question	completely	telling	curious	trust	answer
27	0.035	beautiful	wow	god	story	amazing	woman	women	matter	damn	bless
32	0.033	people	black	racist	claim	considered	folks	drop	bruh	accept	mad
26	0.031	african	africa	north	percent	percentage	west	bit	caucasian	subsaharan	continent
44	0.031	companies	information	data	health	based	sample	info	pay	free	site
47	0.029	irish	german	french	british	english	scandi-navian	scottish	polish	russian	swedish
17	0.029	american	native	indian	america	tribe	mexico	cherokee	elizabeth	megan	claim
41	0.028	father	parents	mother	siblings	biological	passed	grand-parents	child	children	birth
22	0.028	test	government	lie	ad	bs	clone	crime	police	evidence	inaccurate
18	0.026	guys	person	girl	channel	lmao	bro	❤️	vid	reaction	dog
34	0.024	feel	makes	kids	omg	started	sense	hard	cry	baby	laugh
16	0.024	time	told	money	expect	ill	spit	ancestry-com	somali	send	tube
49	0.022	genetic	genes	human	population	common	heard	difference	amount	exact	wtf
48	0.021	white	identical	twins	literally	crazy	nigerian	finnish	confused	noticed	hilarious
28	0.021	hair	eyes	pretty	blonde	blue	red	gorgeous	makeup	alike	green
37	0.021	mixed	race	mexican	culture	latinos	hispanic	roots	latin	term	latina
35	0.020	found	day	ago	hope	happy	sad	months	knowing	weeks	meet
36	0.020	family	adopted	generations	tree	connection	finding	genealogy	figure	lies	records
10	0.019	europe	region	northern	ireland	england	germany	western	britain	viking	russia
33	0.019	blood	ethnicity	chinese	korean	japanese	china	pure	vietnamese	mongolian	korea
45	0.019	dad	mom	born	super	xd	mum	bio	die	josh	es
15	0.019	asian	south	asia	central	east	filipino	india	polynesian	philippines	southeast
21	0.019	country	spanish	spain	iberian	language	portuguese	france	portugal	peninsula	spaniards
29	0.018	ancestors	history	learn	slave	recently	descendants	land	past	slavery	trade
6	0.017	😂	reason	word	stuff	type	remember	haha	truth	earth	jesus
46	0.017	european	speak	southern	majority	broadly	boring	disap-pointed	diverse	typical	decent
42	0.017	arab	origin	greek	proud	turkish	turks	ancient	egyptian	iran	ethiopian
4	0.017	lol	stop	totally	left	wont	eli	thumbnail	scared	theory	smile
13	0.017	live	son	idea	balkan	de	moved	chance	la	usa	uk
19	0.017	world	life	understand	care	rest	mentioned	school	racism	study	planet
40	0.017	share	cousin	related	relatives	match	close	husband	names	lost	uncle
5	0.016	called	real	wrong	explain	yeah	taking	looked	glad	dude	actual
25	0.016	jewish	surprised	jews	nationality	ashkenazi	muslim	religion	israel	forget	boy
2	0.016	theyre	friend	cute	arent	married	thinking	couple	daysha	apparently	huge
20	0.016	guess	true	read	comments	hate	stupid	million	average	random	😂😂
39	0.015	sister	brother	hey	grand-mother	trace	grandfather	line	maternal	chromo-some	haplogroup
14	0.014	italian	italy	wife	roman	empire	honestly	single	😂😂	gay	eugene
23	0.012	talk	fuck	sounds	background	future	hear	voice	music	fascinating	knowledge
3	0.012	middle	eastern	east	means	idk	mediter-ranean	hand	category	meant	finger

ID	γ	Word 1	Word 2	Word 3	Word 4	Word 5	Word 6	Word 7	Word 8	Word 9	Word 10
38	0.011	happened	shit	ppl	body	fake	alot	bullshit	dumb	social	anymore
11	0.010	funny	basically	neanderthal	hell	ha	youll	joke	bitch	bunch	special
30	0.010	lot	shocked	puerto	indigenous	descent	realize	extremely	brazilian	break	considered
7	0.010	science	weird	eat	age	food	picture	phil	dan	sun	dr
24	0.009	skin	dark	brown	color	light	features	darker	fair	tan	colour
50	0.009	heritage	mine	similar	bantu	simply	late	sort	experience	view	main
9	0.008	half	dominican	islands	rican	caribbean	cuban	cuba	taino	euro	rico
12	0.007	grandma	migrated	centuries	turkey	greece	identify	caucasus	lucky	saudi	settled
1	0.001	#marialeen-clases	mine	late	bit	change	bad	shouldnt	telling	excited	break
