# Supplementary material for: Direct-to-Consumer Genetic Testing on Social Media: Topic Modeling and Sentiment Analysis of YouTube Users' Comments
Source: JMIR Infodemiology. 2022 Sep 15;2(2):e38749. doi: 10.2196/38749 (PMC10014090; doi:10.2196/38749)
Supplement: Multimedia Appendix 4 [file infodemiology_v2i2e38749_app4.pdf]

Multimedia Appendix 4. Word Frequency Analysis Results.

The word frequency analysis reveals the total number of occurrences of a word found in all collected comments. The 100 most frequent words of our direct-to-consumer (DTC) genetic testing-related comments dataset are shown in Figure MA4-1. The size of the words in the word cloud depicts their frequency.

Figure MA4-1. Word cloud of 100 most frequent words.

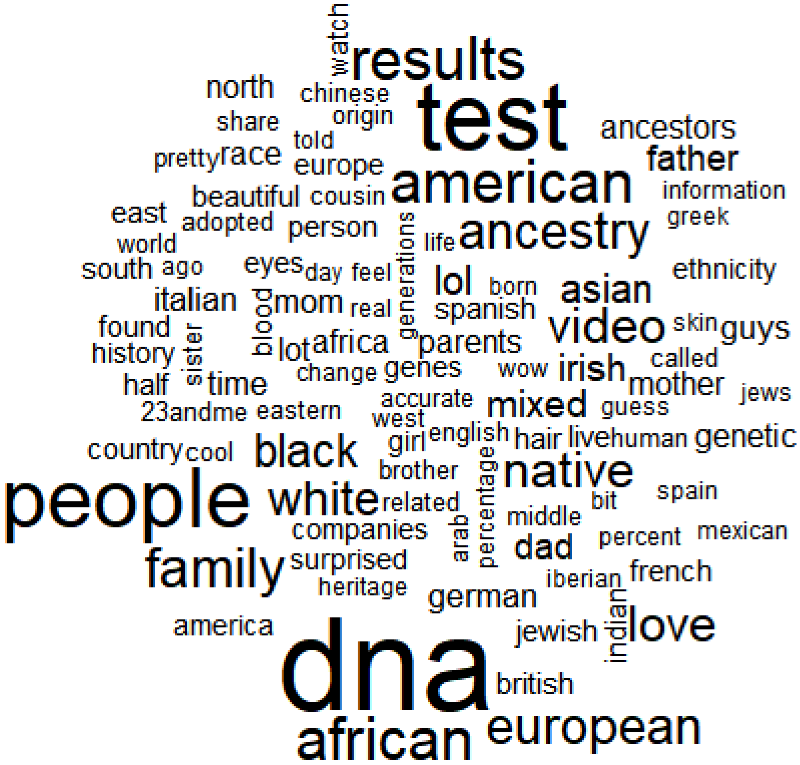

Further, Table MA4-1 lists the 100 most frequent words in descending order by frequency (n). The top three words are DNA (n=15,702), test (n=10,902), and people (n=9259). Frequency reduces quickly for the first 100 words, with the 100th word, told (n=1050), having only approximately 5% of the occurrences of the most frequent word. In total, our dataset includes 46,861 unique words. However, 28,310 of these only occur once, and an additional 13,103 have less than 10 occurrences.

Table MA4-1. 100 most frequent words from comment analysis

Rank	Word	Frequency (n)
1	dna	15,702
2	test	10,902
3	people	9259
4	african	6268
5	results	6178
6	american	6139
7	family	5252

8	european	5142
9	ancestry	5015
10	video	4794
11	love	4751
12	native	4665
13	white	4489
14	black	4203
15	lol	3469
16	asian	3276
17	irish	3177
18	mixed	2984
19	dad	2932
20	father	2782
21	mom	2639
22	time	2560
23	guys	2554
24	german	2548
25	italian	2448
26	ancestors	2344
27	lot	2269
28	africa	2244
29	parents	2228
30	mother	2184
31	north	2170
32	genetic	2130
33	race	2108
34	spanish	2066
35	person	2042
36	jewish	2040
37	country	2032
38	indian	2030
39	east	2019
40	half	1987
41	south	1959
42	found	1955
43	beautiful	1953
44	french	1936
45	live	1917
46	british	1909
47	hair	1854
48	watch	1815
49	blood	1796

50	europe	1775
51	companies	1727
52	girl	1697
53	ethnicity	1689
54	surprised	1673
55	history	1671
56	genes	1650
57	america	1607
58	eyes	1589
59	middle	1526
60	called	1503
61	sister	1480
62	share	1441
63	23andme	1425
64	brother	1424
65	english	1423
66	eastern	1399
67	pretty	1390
68	chinese	1379
69	related	1363
70	arab	1358
71	origin	1345
72	world	1313
73	human	1297
74	cool	1279
75	adopted	1270
76	cousin	1267
77	skin	1253
78	spain	1251
79	feel	1238
80	information	1221
81	guess	1219
82	percent	1210
83	heritage	1204
84	iberian	1174
85	day	1172
86	west	1170
87	percentage	1164
88	wow	1118
89	life	1117
90	mexican	1115
91	ago	1101

92	bit	1083
93	greek	1077
94	real	1077
95	jews	1069
96	born	1060
97	generations	1060
98	change	1057
99	accurate	1052
100	told	1050
